# Intravenous Infusion of Nucleated Peripheral Blood Cells Restores Fertility in Mice with Chemotherapy-Induced Premature Ovarian Failure

**DOI:** 10.3390/biomedicines6030093

**Published:** 2018-09-15

**Authors:** Abdeljabar El Andaloussi, Prosper Igboeli, Amero Amer, Ayman Al-Hendy

**Affiliations:** 1 Departments of Obstetrics and Gynecology, University of Illinois at Chicago, 820 South Wood Street, Chicago, IL 60612, USA; pigboe2@UIC.EDU (P.I.); aalhendy@uic.edu (A.A.-H.); 2 Meharry Medical College, Nashville, TN 37011, USA; ameroamer1980@yahoo.com; 3 Georgia Reagent University, Augusta, GA 30909, USA

**Keywords:** premature ovarian failure (POF), PBMC (Peripheral blood mononucleated cells), chemotherapy, cancer, ovary

## Abstract

Cancer treatment with specific chemotherapeutic agents has been well documented to have an adverse impact on female fertility leading to premature ovarian failure (POF). The objective of this study is to investigate if chemotherapeutic induced POF can be reversed by the infusion of autologous nucleated peripheral blood cells (PBMC). To reach our goal, mice were treated with a single intraperitoneal injections of busulfan and cyclophosphamide to induce POF. This was followed by transfusion of PBMC. The ovarian morphology and functional parameters were monitored by radioimmunoassay, real-time PCR, immunofluorescence and immunohistochemistry analysis. Our study showed that chemotherapy (CTX) protracted estrous cycle period and repressed E2 production. In addition, CTX decreased the expressions of steroidogenesis markers, *CYP-17* synthesis, *StAR* (steroidogenic acute regulatory protein), and Connexin-43 protein expression in the ovarian follicles. We also observed reduced numbers and sizes of the primordial and primary follicles in CTX-treated mice compared to untreated controls (*p* < 0.05). When both CTX and untreated control groups were stimulated with gonadotrophin, the control group produced ten times more ova than the CTX group. Finally, the treatment of premature ovarian failure induced by CTX with autologous PBMC transfusion resulted in over-expression and a statistically significant increase in several stem cell markers and restoration of fertility. Infusion with PBMC in CTX further decreased the estrous cycle length by 2.5 times (*p* < 0.01). We found that transfusion of autologous PBMC to mice with chemotherapy induced POF was very effective at restoring fertility. These results are similar to other studies using bone marrow derived mesenchymal stem cells.

## 1. Introduction

Approximately 600,000 American women are diagnosed with cancer each year, of whom 8.0% are of pre-reproductive age [1]. Although cancer incidence has been increasing annually, earlier detection and more effective treatments have reduced mortalities by approximately 1.0% per annum since the 1990s [2]. Chemotherapy (CXT) is the most common treatment modality used in cancer patients followed by surgery, radiotherapy, and other more specialized treatment [3]. Premature ovarian failure (POF) is a common long-term consequence of CTX and radiotherapy [4,5]. The risk of gonadal damage is usually dose and age dependent. As survival rates for young female cancer patients continue to improve, protection against iatrogenic POF caused by CTX and preserving future fertility become central in planning, management and outcome [6]. The American Society of Clinical Oncology and the American Society for Reproductive Medicine have issued guidelines stipulating that the potential adverse effects of cancer treatment on fertility, as well as fertility preservation options, be presented to patients in the initial stages of counseling, planning and treatment [7]. The existing approaches aimed at preserving fertility in female cancer survivors include: (a) protection of ovarian follicles with gonadotropin releasing hormone agonist (GnRH-a) concurrently administered with CTX; and (b) ovarian tissue, ova or embryo freezing prior to CTX. Some of these interventions have shown inconsistent outcome, some require invasive surgical procedures to permit the recovery of ovarian tissue, and other procedures, like oocyte cryo-preservation, involve ovulation induction protocols, followed by ultrasound guided oocyte retrieval and subsequent cryo-preservation for in vitro fertilization (IVF) later in life. The cost of these procedures can become quite substantial and unaffordable for many patients. Furthermore, procedures like oocyte cryo-preservation are not considered achievable options for young pre-pubertal girls. Clearly, less invasive and cost-effective alternative approaches are highly desirable for fertility preservation in both pre-pubertal and reproductive age female cancer survivors. Recent work from our group and others suggested that fertility preservation can be achieved by transplantation of adult stem cells from heterologous and autologous sources into female cancer survivors [8,9].

Anti-cancer therapeutic agents such as cyclophosphamide, Ifosfamide, Nitrosoureas, Busulfan, Melphalan, Mustagene, Chlorambucil, Dacarbazine, Procarbazine, and Cisplatinum are alkylating agents and their use are associated with gonadal damage [10,11]. More specifically, these chemotherapeutic agents have been shown to destroy ovarian follicles [12,13]. There is a controversy as to the particular cell type(s) in the ovarian follicles that are affected by CTX. Some proponents postulate that chemotherapeutic agents appear to directly target oocytes in primordial and primary follicles for apoptotic destruction [14,15]. It is well established that rapidly dividing cells are known to be more sensitive to the cytotoxic effects of alkylating agents than are cells at rest. This makes steroidogenic cells of the follicle the likely target for CTX action [4,6,16]. Therefore, it is highly unlikely that postnatal oocytes which are at the dictyate state of arrest and do not undergo mitosis are susceptible to apoptotic destruction by alkylating agents used for cancer chemotherapy [17,18]. We hypothesized that somatic cells (i.e., theca or granulosa cells or both) rather than oocytes are the sensitive cells to cancer therapeutic agents. Mitosis among presumptive oocytes occurs prenatally and all postnatal oocytes remain at the dictate state of meiotic maturation until recruitment and exposure to an LH surge. The objectives of this study were: (1) to determine the specific cells in the follicles targeted by CTX agents and the ultimate effect(s) it has on oocyte maturation and quality; and (2) to determine the efficacy of syngeneic blood borne stem cells to restore function to CTX-damaged ovaries. The later objective will serve as a prelude to assessing the possibility of rejuvenating fertility with autologous adult stem cells among cancer survivors.

## 2. Materials and Methods

### 2.1. Animals

Adult female SV-129 mice, 6–7 weeks of age and weighing between 25–35 g, were purchased from Charles River Co. (Wilmington, MA, USA). Animals were housed in groups of four in polyethylene cages and allowed to acclimatize to the animal facility environment for at least one week prior to initiation of studies. Animals were maintained in an environmentally controlled room with 12 h light: 12 h dark cycle (lights on at 6:00 a.m.), 22 °C and humidity range of 50% to 60% and allowed ad libitum access to commercial pelleted mouse chow and water. All animal work described in this manuscript was approved by the institutional animal care and utilization committee of Georgia Regent University (Protocol 2014-0646, 29 May 2014) Acclimatized mice were randomly assigned to a treatment or control group. Treatment consisted of single Intraperitoneal (IP) injections of Busulfan (12 mg/kg) dissolved in DMSO and cyclophosphamide (120 mg/kg) dissolved in saline to 6 weeks old mice. This combination is hereafter referred to as chemotherapy (CTX). Mice in the control group were similarly injected with DMSO and saline (vehicle controls). Seven days post injection with CTX, mice in the CTX and control group were divided into 3 groups (A–C). Mice in group A were subjected to daily vaginal lavage according to the method of Aliagas et al. [19] for the determination of the effect of CTX on stages and overall length of the estrous cycle. Mice in groups B and C were each subjected to ovarian hyperstimulation with a single IP injection of 5 IU of equine gonadotropin (EG). Forty eight h after EG injection, a period corresponding to Proestrus, the stage of the estrous cycle characterized by enhanced folliculogenesis and estrogen biosynthesis, mice were anesthetized with isoflurane and the abdominal contents exposed via mid-ventral laparotomy to facilitate blood collection from the inferior vena cava and excision of ovaries.

### 2.2. Blood and Serum Preparation

Blood samples from CTX-treated or control mice were pooled at the rate of 3 mice/treatment/tube to give final sample size of 6 for CTX-treatment or control group and placed on ice until centrifuged at 4 °C at 1500× *g* for 10 min. Sera were harvested and stored frozen at −20 °C until analyzed for E2 and luteinizing hormone (LH) contents. Excised left ovaries (*N* = 6/group) were fixed in 4% buffered formalin for 24 h prior to being stored in 70% ethanol until paraffin blocked and sectioned. Ovarian tissue sections were subsequently subjected to H&E to assess the distribution of ovarian follicle developmental stages and antral follicle size distribution. Sections were also subjected to immunohistochemical (IHC) staining to assess the activities of steroidogenesis acute regulatory (*StAR*) protein, steroidogenic enzymes and the integrity of granulosa cell gap junction protein, respectively. The Equine gonadotropin-stimulated CTX-treated and control mice (*N* = 12/group) in group C were injected IP with 5 IU of human chorionic gonadotropin (hCG) 48 h post EG and either sacrificed 16 h later (*N* = 6/group) to assess ovulation rates and the meiotic maturational stages of ovulated ova or subjected to mating (*N* = 6/group).

### 2.3. Isolation of PBMC

Blood samples were collected from inferior vena cava of adult donor SV129 female mice under anesthesia induced with isoflurane. Subsequently, mononuclear cells (PBMC) were harvested post gradient centrifugation at 800× *g* for 15 min at 4 °C with Ficoll-Paque Plus (Amersham Biosciences/GE Healthcare, Piscataway, NJ, USA). Nucleated peripheral blood cells were subsequently washed by centrifugation in PBS as described above and re-suspended in PBS to a final concentration of 2× 10^7^/mL. The PBMC were infused per tail vein injection 2 weeks post CTX treatment.

### 2.4. Apoptosis Tunel Assay

The apoptotic signal was determined by TUNEL assay using a commercially available kit (In Situ Cell Death Detection Kit, fluorescein, Roche, Indianapolis, IN, USA), following manufacturer recommendations. Briefly, the fixed cells on the slides were washed three times for 5 min with PBS- and permeabilized with 0.1% (*v*/*v*) Triton X-100 containing 0.1% (*w*/*v*) sodium citrate for 2 min on ice. Samples were then incubated in 50 μL of TUNEL reaction mixture for 1 h at 37 °C in a dark and humidified atmosphere. Slides were washed three times with PBS- and cover slips were mounted using mounting medium (VECTASHELD^®^, Vector Laboratories, and Burlingame, CA, USA). Positive TUNEL GFP staining was observed under a fluorescence microscope (TE2000U, Nikon, Tokyo, Japan) using the B-2A filter (450–490 nm excitation filter, 505 nm dichroic mirror, 520 nm band pass filter).

### 2.5. Ovulation Rate and Maturational Stages of Ovulated Ova

Oviducts excised from mice subjected to ovulation induction protocol were flushed with PBS containing 0.1% hyaluronidase (Sigma Chemical Co., St. Louis, MO, USA) pre-equilibrated at 37 °C. Recovered cumulus masses (ovulated eggs invested in cumulus tissue) were allowed to incubate in the presence of hyaluronidase for 15 min. The freed ova were then retrieved and placed in hyaluronidase-free PBS. Mean number of ovulated eggs/mouse was determined by counting all the eggs post hyaluronidase treatment/paired oviduct/treatment group divided by the 6 (number of mice/treatment). Ovulated eggs were staged as mature (MII meiotic maturational stage) if each ovum contained a single polar body in the perivitelline space. Oocytes were classed as immature and staged as being in metaphase I meiotic stage of maturation (MI) if they lacked both a GV in the vitellus and a polar body in the perivitelline space or staged as being in GV meiotic maturational stage if they had a GV in the vitellus but lack a polar body in the perivitelline space.

### 2.6. Determination of Ovarian Follicle Reserve

Five micron sections of ovaries from CTX and control mice were cut and subjected to H&E staining in the Meharry Human Tissue Acquisition and Immunohistochemistry Shared Resource Core laboratory (HTAHICL). We stained every 10th slide from the entire ovarian paraffin block. Stained sections were evaluated antral follicles versus corpora lutea based on the criteria of Pederson and Peters [20]. The diameter of antral follicles and structural morphology were classified as healthy or atretic Follicles. All section were examined by an assessor who was blinded for experimental group assignment to avoid any observer bias.

### 2.7. Immunohistochemistry Staining and Image Analysis

Sections of ovaries (5 µm thick) from CTX and control mice were subjected to indirect immunohistochemical (IHC) staining in the Vanderbilt University IHC Core laboratory. Sections were stained for steroidogenesis regulatory protein/enzyme expressions and Connexin-43 to evaluate whether CTX induced ovarian failure via apoptotic activities by Tunel Assay, alteration of steroidogenesis enzyme expressions and integrity of gap junction among granulosa cells, respectively. A total of 50 Images for ovarian sections (*n* = 10) were acquired with a Nikon TE2000-E inverted microscope, using a 10× and 20× objective with numerical aperture (NA) 0.30 and 0.75 NA, respectively. Semi quantitative analysis of mean intensities of control and CTX for *StAR* (ab58013, *d* = 1/500), CYP17 (Ab134910, *d* = 1/500), aromatase, and Connexin-43 (ab11370, *d* = 1/500) stained sections were performed in conjunction with peroxidase-based (DAB) immunostaining systems, using NIS Elements Advanced Research Software (Nikon, Melville, NY, USA). Ten to 17 regions of interest (ROI) were outlined on images obtained from each condition using the NIS Elements software. The background intensity was subtracted from the ROI, and an intensity threshold was set and kept constant for all images analyzed. Mean intensity per square micrometer area was calculated by dividing the mean intensity units by the area of outlined regions.

### 2.8. mRNA Isolation and cDNA Preparation

Total RNA was extracted from the ovaries of CTX-treated and control mice using the RNeasy kit (QIAGEN, Germantown, MD, USA). RNA was further mixed with gDNA elimination buffer to remove genomic DNA contaminants according to the manufacturer’s instruction. The content of each RNA extraction tube was gently mixed, briefly centrifuged with a Picofuge (Stratagene, La Jolla, CA, USA) at ambient temperature and incubated at 42 °C for 5 min and then chilled on ice for 1 min. Equal volume (10 uL) of master mix was added to each genomic DNA elimination mixture and mixed gently with a pipettor. Each mixture was incubated at 42 °C for 15 min and immediately heated to 95 °C for 5 min to stop the reaction. Subsequently, 91 µL of H_2_O supplied in the kit was added to each 20 µL cDNA synthesis reaction tube and mixed very well before being stored frozen at −20 °C until analyzed. Primers sequences are summarized in Table S1.

### 2.9. Real Time PCR

cDNA samples were thawed, centrifuged with a Picofuge (Stratagene) for 15 sec prior to being maintained at 4 °C and used for RTPCR. 2× SA Bioscience RT2 qPCR master mix (1.350 mL) was mixed with 102.0 µL of each cDNA sample and 1.248 mL of kit supplied water in a 5 mL tube. After careful removal of PCR array plate (PAMM-054, SA Biosciences, Baltimore, MD, country) from a sealed bag, 25 µL of each experimental cocktail were added to each well with an 8 channel pipettor. A two-step cycling program was performed using myiQ5 cycler (BioRad, Hercules, CA, USA) in which the first step was for 10 min at 95 °C and the second step was for 40 cycles (15 sec at 95 °C and 60 °C for 1 min each).

### 2.10. Radioimmunoassay

Sera were analyzed for E2 using RIA method previously validated in our laboratory [21]. The sensitivity of E2 assay was 2 pg/tube and the intra- and inter-assay coefficients of variation (CVs) were 4.9 and 10.8% respectively. Sera were also analyzed for LH by the above mentioned method in West Virginia Endocrine Core laboratory.

### 2.11. Statistical Analysis

Data on the effect of CTX on estrous cycle length, antral follicle size, apoptosis, expression of steroidogenesis enzymes, serum E2 and LH concentrations, Connexin-43 expression and ovulation rate were compared with unpaired *t*-test. The preponderance of within ovary apoptotic signal between pre-antral and antral follicles was determined with paired *t*-test. Data on the percentage of mature eggs ovulated were analyzed by Chi-square. Data on the effect of PBMC on estrous cycle length and litter size of CTX-treated mice were analyzed by One-Way ANOVA and the differences among means were tested with orthogonal contrasts.

## 3. Results

### 3.1. Effect of Chemotherapy Treatment on Estrous Cycle and Hormone Production

**Figure 1:**
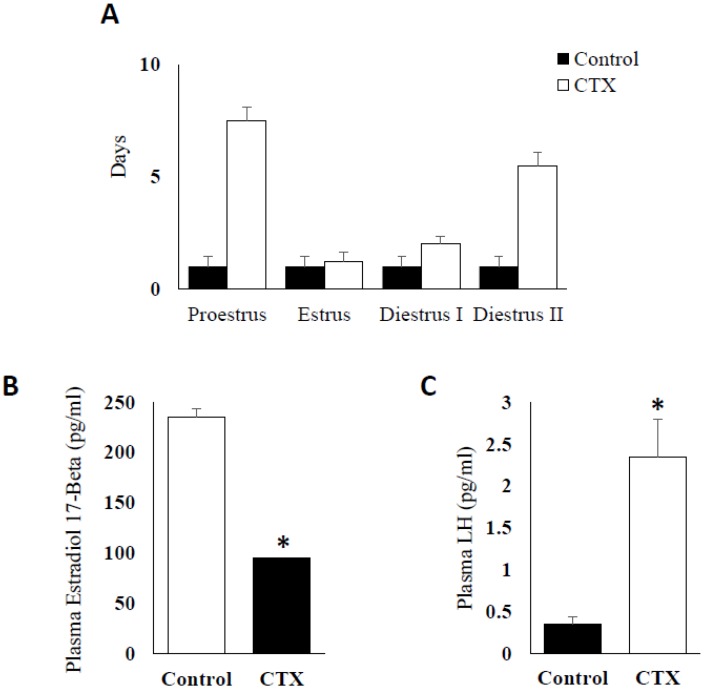
Effect of chemotherapy treatment on estrus cycle and steroidogenesis. (**A**) Effect of CTX (Chemotherapy) on estrus cycle length; (**B**) comparison of plasma estradiol 17-β concentrations presented in pg/mL between CTX-treated mice group and control group at Proestrus stage (* *p* < 0.05); (**C**) plasma LH (Luteinizing hormones) levels presented in pg/mL and compared between CTX-treated mice group and control group at Proestrus stage (* *p* < 0.05).

Mice treated with CTX sustained a significant increase in the length of estrous cycle up to 18 days cycle versus the normal four days cycle observed in control mice. The cycle was prolonged in all the phases but particularly seen in proestrus and diestrus phases (Figure 1A) (*p* < 0.005). The chemotherapy treatment also exerted significant effects on serum reproductive hormone levels with reduction in Estradiol-17β (Figure 1B) and marked increase in plasma LH values (*p* < 0.05) (Figure 1C), two markers required for active ovary with normal steroidogenesis and the control of estrus cycle phases.

### 3.2. Chemotherapy Induces Apoptosis of Granulosa Cells

**Figure 2:**
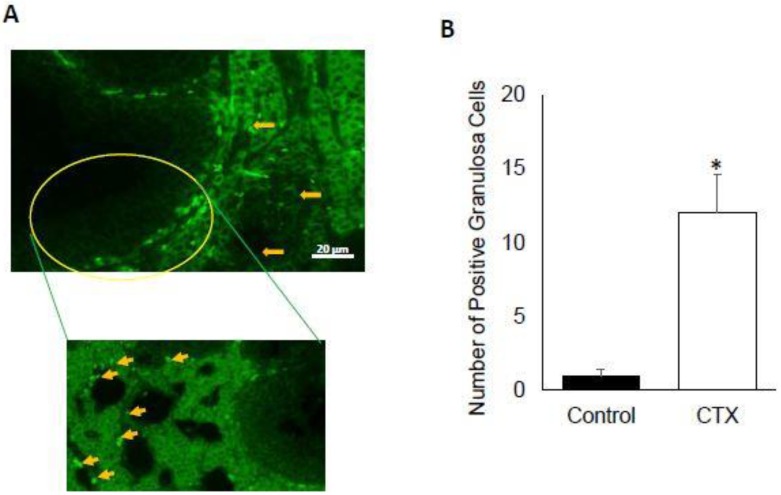
Impact of chemotherapy on apoptosis in murine granulosa cells. (**A**) Tunel staining (green) on ovary cut section, positive staining showed by yellow arrows. Data supported by high magnification of the circle area; (**B**) graph bar of tunel positive granulosa cells number compared to the matched control (* *p* < 0.05).

Chemotherapeutic agents are expected to induce cell death in rapidly proliferating cells. To explore that, we used tunel assay to quantify the number of apoptotic granulosa cells in the ovarian follicles of CTX-treated versus vehicle control animals. The analysis of granulosa cells subjected to tunel assay using fluorescence microscope, showed the presence of significant difference of positive cells for tunel in CTX-treated mice compared to the untreated control (Figure 2A,B) (*p* < 0.001). The number of tunel-positive cell for was increased in ovarian sections from CTX-treated mice group (13.33 ± 1.24) compared to the control group (2.33 ± 0.33) (*p* < 0.001).

### 3.3. Alteration of Expression of Steroidogenesis and Follicular Gap Junction Genes

**Figure 3:**
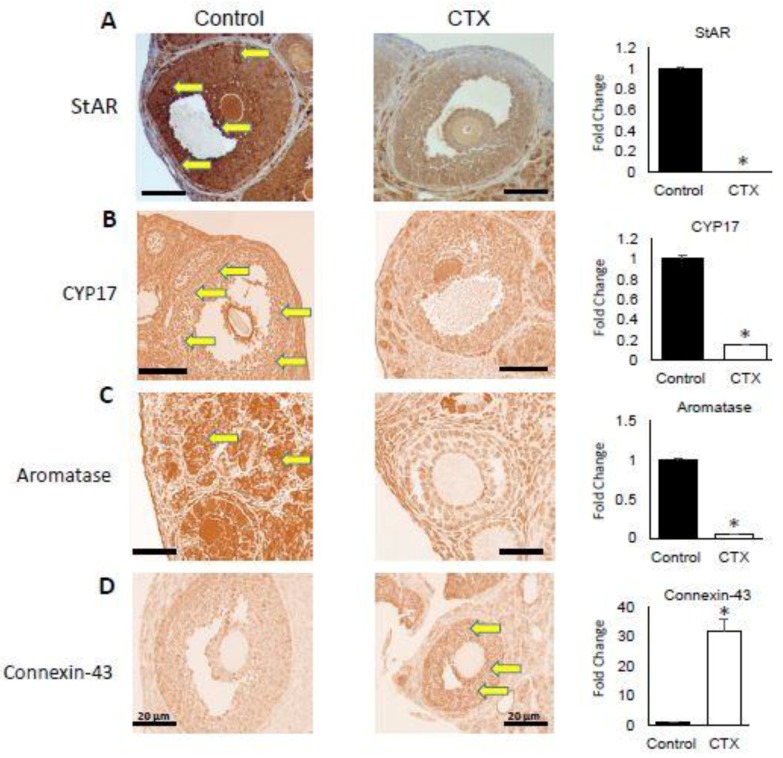
Steroidogenesis markers expressions suppressed by chemotherapy. Cross-section of the ovaries in both the CTX-treated group on the left side and control group on the right side analyzed by immunohistochemistry staining for specific targets involved in the ovarian function supported by fold change (on the right side) for statistical analysis: (**A**) *StAR* (steroidogenic acute regulatory protein), (**B**) *CYP-17*, (**C**) aromatase and (**D**) Connexin-43. For the four markers * *p* < 0.05. Some slides contain yellow arrows indicating the positive staining in granulosa cells. A Total of 10 sections was analyzed for each markers.

To study the effect of CXT treatment on various steps of steroidogenesis as well as follicular structure, we selected the following markers: *StAR, CYP-17, aromatase and connexin-43*. The analysis of the expression of these genes was evaluated via immunohistochemistry. The *StAR* protein is responsible of cholesterol translocation from the outer to the inner mitochondrial membrane in steroidogenic cells and is also the rate limiting step in steroid hormone formation [22]. We found that CTX treatment significantly inhibited the expression of *StAR* at protein level (Figure 3A) (*p* < 0.05). Another key steroidogenesis regulatory enzyme is *CYP-17*, localized to the endoplasmic reticulum and playing a major role in the steroidogenic pathway that produces progestins, mineralocorticoids, androgens, and estrogens. The expression of *CYP-17* was also significantly downregulated by CTX treatment when compared to untreated controls (Figure 3B) (*p* < 0.05). In addition, aromatase expression was significantly abrogated by CTX compared to the control (Figure 3C) (*p* < 0.05). On the other hand, connexin-43 gene expression was elevated in the ovary of mice group treated with CTX versus control group consistent with abrogated follicular development (Figure 3D) (*p* < 0.05).

### 3.4. Chemotherapy Decreases Oocytes Production after Ovarian Stimulation

**Figure 4:**
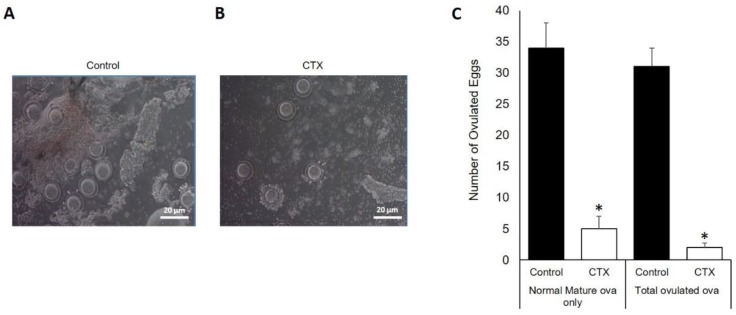
Chemotherapy effects on ovarian stimulation and follicular development. Microscopic illustration of mouse oocytes from control (**A**) and CTX (**B**) exposed mice; (**C**) graph bar summarize the number of ovulated ova after gonadotropins injection in both the CTX-treated group of mice and the control group (* *p* < 0.05).

To evaluate oocyte production ability of animals treated with CTX and its impact on the ovarian reserve, these animals were stimulated with gonadotropin as described in the methodology section. The analysis of ovarian sections by microscopy (Figure 4A,B) showed that under CTX treatment the total numbers of mature and ovulated oocytes were significantly decreased compared to untreated control (Figure 4C) (*p* < 0.05). The control group were found to produce more than 10 times the number of ovulated matured oocytes and seven times total ovulated oocytes than CTX group (Figure 4C). This observed response is due to limited response to gonadotropin stimulation in CTX-treated mice.

### 3.5. Effect of CTX on Ovarian Follicle and Corpora Lutea Numbers

**Figure 5:**
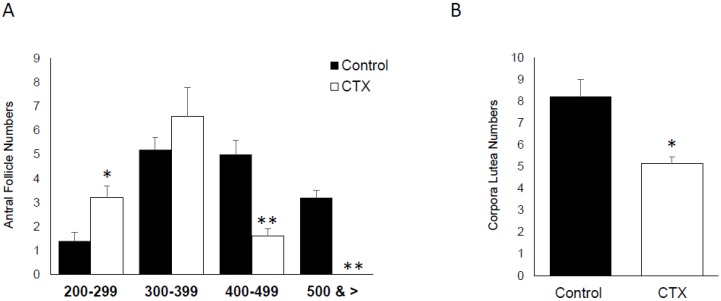
Effect of chemotherapy on antral follicle and corpora lutea numbers. (**A**) Graph bar of antral follicle number in CTX treated group versus untreated control group (* *p* < 0.05, ** *p* < 0.005); (**B**) numbers of corpora lutea formed following ovulated ova in the CTX treated group versus control group (* *p* < 0.05).

The difference between the folliculogenesis dynamic of CTX-exposed versus untreated mice was evaluated for antral follicles and corpora lutea numbers. The number of ovarian follicles, which are hypothalamic/pituitary gland-dependent and represent later stages of folliculogenesis, was divided into four group based on size ranges: 200–299 µm, 300–399 µm, 400–499 µm and above 500 µm. For the first group of 200–299, the total number of antral follicles was significantly induced by CTX treatment (*p* < 0.05). However, this effect of CTX was reversed significantly in the groups of 400–499 and above 500 (Figure 5A) (*p* < 0.05). In addition the number of corpora lutea was significantly reduced in the group of mice treated with CTX (Figure 5B) (*p* < 0.05).

### 3.6. Nucleated Peripheral Blood Cells Infusion Restore Estrous Cycle Length in CXT-Treated Mice

**Figure 6:**
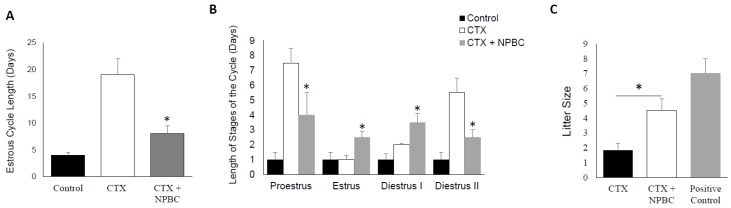
Impact on nucleated peripheral blood cells on estrus cycle and fertility. (**A**) Estrous cycle length presented in days comparing CTX to CTX treated with PBMC versus matched negative control (* *p* < 0.05); (**B**) length of estrus cycle stages presented in days of the three involved mice groups: Control, CTX and CTX treated with PBMC (Peripheral blood mononucleated cells) (* *p* < 0.05); (**C**) effect of PBMC on fertility presented in litter size compared between CTX to CTX treated with PBMC versus matched negative control (* *p* < 0.05).

To rescue the mice ovarian functions, we treated them with intravenous infusion of PBMC as described in the methods section. The effect of this treatment was evaluated at the level of estrus cycle cyclicity. We observed that PBMC reverse the acylic effect of CTX and there was significant reduction in the estrus cycle days by approximately 10 days in the group of mice treated with CTX followed with PBMC when compared to mice group treated with CTX alone (*p* < 0.01) (Figure 6A). The impact of PBMC was detected on all phases of the estrous cycle (Figure 6B).

### 3.7. Nucleated Peripheral Blood Cells Rescues the Negative Effect of CTX on Fertility

To test the effect of PBMC treatment on the ovarian failure induced by CTX, we subjected our experimental groups to mating with age matched male mice and monitored the vaginal mucus plugs. All the mice in the control group exhibited normal fecundity, as evidenced by the presence of mating plug in each animal’s vaginal os, within seven days of co-habitation with males. However, evidence of mating in CTX-treated group and PBS was observed only after day 21. For the mice treated with CTX and PBMC, they mated (sperm plugs visible) within two weeks following PBMC infusion. Importantly, CTX/PBMC treated female mice demonstrated significantly larger litter size and produced overall significantly higher number of pups during same mating period compared to CXT/Vehicle treated control group (*p* < 0.05) (Figure 6C).

### 3.8. Nucleated Peripheral Blood Cells Treatment Promotes Stem Cell Markers in the Chemotherapy-Exposed Ovaries

To gain a further insight into the potential ovary-reparative mechanisms underlying the functional benefits observed following treatment with PBMC, we quantified the expression levels of several genes known to be associated with ovary reparative functions using Q-PCR (Quantitative polymerase chain reaction) as described in the methods section. These included vascular endothelial growth factor A (Vegf-A) [23,24], colony stimulating factor-1 (Csf-1) [25,26], Neurogenic locus notch homolog 4 (Notch4) [27,28], inhibin beta A (INHBA) [29], kit ligand (kit-l) and CD34 [30,31,32]. Interestingly, we found that treatment of mice CTX followed by PBMC intravenous infusion supported significantly high expression levels of all mentioned markers in ovarian tissues compared to CXT/Vehicle treated group (*p* < 0.05) (Table 1).

## 4. Discussion

In this study, CTX did not only affect the ability of treated mice to cycle regularly, it also extended the length of the cycle by 18 days compared to their control counterparts. This observation indicates a repression in E2 synthesis and release imposed by CTX that resulted in protracted changes in the length of the estrous cycle and mostly halts in the proestrus phase [33]. The extension in the length of the estrous cycle among the CTX-treated animals occurred at diestrus II and proestrus phases of the estrous cycles at which increases in serum E2 concentrations are initially apparent and subsequently maximal respectively [34].

The follicles develop through primordial, primary and secondary growing stages (gonadotropin-independent pre-antral follicular growth) before acquiring an antral cavity. At the antral stage, a few of these follicles respond to cyclic gonadotropin stimulation and ultimately reach the pre-ovulatory stage of development, at which stage, they serve as the major source of E2 production [35]. The reduction in serum E2 concentrations observed among CTX-treated animals is a function of the down regulation of *StAR*, *CYP-17* and aromatase enzyme expression. The reduced E2 secretion showed by our data is a reflection and is consistent with the limited number of pre-ovulatory follicles seen in the CTX-treated mice. We therefore contend that the extended duration of diestrus II and proestrus in CTX-treated mice is due to insufficient E2 secretion from the follicles to trigger the progression of the estrous cycle towards estrus in a timely manner. Consistent with the reduction in serum E2 concentrations among CTX-treated mice, ovaries from these animals sustained a higher incidence of follicle losses in all follicle categories as well as reduced antral follicle size compared to their control counterparts. Normal folliculogenesis is characterized by high rates of proliferation and apoptosis within follicles and balances between local ovarian growth factors and circulating hormones [36,37]. In the present study we observed that the number of antral follicles in the ovaries of CTX-treated and control mice exhibiting apoptotic signal were comparable. These observations suggest that growing follicles constitute the most vulnerable group of follicles in the ovary to CTX, reflected by a decrease of ovarian reserve. However, some investigators postulate that chemotherapeutic agents, most of which are alkylating agents, appear to directly target oocytes in primordial and primary follicles for apoptotic destruction [14,15]. This is highly unlikely in view of the fact that postnatal oocytes are non-mitotic cells and therefore not susceptible to apoptotic destruction by cancer chemotherapeutic agents. Dividing cells are known to be more sensitive to the cytotoxic effects of alkylating agents than cells at rest [17,38]. Thus, it has been established that the site for chemotherapy-induced apoptotic damage in the ovary is the granulosa cell, following which, atresia in oocytes in antral follicles due to lack of granulosa cell support might eventually ensue [14,15]. The lack of granulosa cell support may be a function of gap junction maintenance. The complete development of ovarian follicles requires the maintenance of a bi-directional communication between the granulosa cells and the oocyte [39]. The cross talk between oocytes and surrounding follicular (granulosa) cells plays an active role in follicular development, being responsible for the proliferation, development and function of granulosa cells [39]. Ongoing granulosa-oocyte communication is critical for oocyte growth, the development and maturational competence and the maintenance of meiotic arrest [40,41]. Connexin-43, one of the pivotal follicular gap junction proteins is the primary marker for the cell-cell communication channels among granulosa cells [42]. The disruption of this communication is usually associated with pre-ovulatory surge of LH at proestrus. The LH-induced breakdown in communication that corresponds with the resumption of meiosis [43]. We observed a continuous expression of connexin-43, concurrent with significantly low serum concentrations of E2 and LH at proestrus among granulosa cells of CTX-treated mice compared with controls. These observations is a reflection of inhibition of follicular development and of ovulation.

Interestingly, several markers, such as Vegf-A, Csf-1, Notch4, inhba, kit-l and CD34, Notch4, inhibin beta A (INHBA), kit ligand (kit-l) and CD34 are induced due to the fact that the implanted PBMC home to the injured ovary and provide homeostatic support, likely via secreting various growth factors, which revive the local stem cell population that in turn proceed to differentiate to ovarian somatic cells. Regardless of the exact mechanism, it is clear that implantation of PBMCs have supported various functions (folliculogenesis, hormone production, and fertilizable egg production) in the chemotherapy-damaged ovaries. These findings provide important translational data for fertility preservation in young female cancer survivors or young women who are exposed to chemotherapy at early age for various indication [44].

The reduction in the length of estrous cycle and the restoration of fecundity and fertility via restoration of function to the ovaries of CTX-treated mice after PBMC infusion are promising findings and suggest that cell-based therapy for CTX-induced infertility is feasible. It has been established that mesenchymal stem cells (MSCs) are present in peripheral blood and are morphologically fibroblast-like cells that possess the ability to self-renew and differentiate into tissues of mesodermal origin such as the ovary [45]. This indicates cellular pluripotency and suggests that MSCs are responsible for the normal turnover and maintenance of adult mesenchymal tissues [46]. The findings of our study suggest that fertility restoration in cancer chemotherapy-treated patients of reproductive age can be affected via autologous transfusion of PBMC harvested prior to the delivery of chemotherapy. This process is cost effective, minimally invasive, low risk and does not necessitate the laborious isolation and expansion of MSCs.

## Figures and Tables

**Table 1:** Ovarian expression of stem cell markers evaluated by real-time PCR and compared between mice group treated with PBMC and untreated controls (CTX only and negative control) (*p* < 0.05).

Gene	Fold Change	Function	References	*p* Value
*CD34*	26.8	Mediate the attachment of stem cells to the bone marrow extracellular matrix or directly to stromal cells. Act as a scaffold for the attachment of lineage specific glycans, allowing stem cells to bind to lectins expressed by stromal cells or other marrow components. (enhance stem cell activity)	[33,34,35]	<0.05
*Csf1*	60.2	The protein encoded by this gene is involved in development of the placenta. It plays a role in fertility and pregnancy. (blood cell differentiation)	[28,29]	<0.05
*Inhba*	2.2	Beta A subunit form a pituitary FSH secretion inhibitor effectively regulating gonadal stromal cell proliferation negatively but when joined with Beta B subunit it stimulate FSH secretion	[32]	<0.05
*Kitl*	24.7	Play a role in cell migration, cell-cell adhesion and augment proliferation of myeloid progenitors in bone marrow culture. (blood cell differentiation)	[33,34,35]	<0.05
*Notch4*	20.0	Functions as a receptor for membrane-bound ligands to regulate cell fate determination by working as a signaling network between adjacent cells. Regulate branching morphogenesis in the developing vascular system.Increase blood supply to stem cells	[30,31]	<0.05
*Vegfa*	143.1	Encodes a protein that mediate increased vascular permeability, including angiogenesis, vasculogenesis and endothelial cell growth and in effect inhibits apoptosis. Increase blood supply to stem cells	[26,27]	<0.05

## Supplementary Materials

Supplementary materials can be found at http://www.mdpi.com/2227-9059/6/3/93/s1.
