# Supplementary material for: Intravenous Infusion of Nucleated Peripheral Blood Cells Restores Fertility in Mice with Chemotherapy-Induced Premature Ovarian Failure
Source: Biomedicines. 2018 Sep 15;6(3):93. doi: 10.3390/biomedicines6030093 (PMC6163893; doi:10.3390/biomedicines6030093)
Supplement: Supplementary file 1 [file biomedicines-06-00093-s001.pdf]

Table S1: Primers sequence

Gene	Primer name (5' –> 3')	
**CD34**	Forward Primer	CTACAACACCTAGTACCCTTGGA
	Reverse Primer	GGTGAACACTGTGCTGATTACA
**Csf1**	Forward Primer	TGGCGAGCAGGAGTATCAC
	Reverse Primer	AGGTCTCCATCTGACTGTCAAT
**Inhba**	Forward Primer	CCTCCCAAAGGATGTACCCAA
	Reverse Primer	CTCTATCTCCACATACCCGTTCT
**Kitl**	Forward Primer	AATCCTCTCGTCAAAACTGAAGG
	Reverse Primer	CCATCTCGCTTATCCAACAATGA
**Notch4**	Forward Primer	TGTGAACGTGATGTCAACGG
	Reverse Primer	ACAGTCTGGGCCTATGAAACC
**Vegfa**	Forward Primer	AGGGCAGAATCATCACGAA
	Reverse Primer	AGGGTCTCGATTGGATGGCA
